# Percutaneous Liver Biopsies Guided with Ultrasonography: A Case Series

**DOI:** 10.5812/iranjradiol.13184

**Published:** 2013-08-30

**Authors:** Emin Cakmakci, Kosti Can Caliskan, Omer Naci Tabakci, Mehmet Tahtabasi, Zeki Karpat

**Affiliations:** 1 Department of Radiology, Sisli Etfal Training and Research Hospital, Istanbul, Turkey

**Keywords:** Biopsy, Specimen Handling, Ultrasonography

## Abstract

**Background:**

Although liver biopsy is an easy procedure for hospitalized patients and outpatients, some complications may occur.

**Objectives:**

To evaluate the efficiency, complications, safety and clinicopathological utility of ultrasonographic-guided percutaneous liver biopsy in diffuse liver disease.

**Patients and Methods:**

In our retrospective study, we evaluated ultrasound-assisted needle biopsies that were performed in outpatients from October 2006 to July 2010. The liver biopsies were performed following one-night fasting using the tru-cut biopsy gun (18-20 gauge) after marking the best seen and hypovascular part of the liver, distant enough from the adjacent organs.

**Results:**

A total of 1018 patients were referred to our radiology department. Most of the patients had hepatitis B (60.6%). The biopsy specimens were recorded and sent to our pathology department for histopathological examination.

**Conclusion:**

According to the results of our series, percutaneous liver biopsy using the tru-cut biopsy gun guided by ultrasonography can be performed safely. We resolve that routine ultrasound of the puncture site is a quick, effective and safe procedure. The complication rate is very low. The US-assisted percutaneous liver biopsy should be used for all cases.

## 1. Background

Since the 1980’s, the number of image-guided percutaneous procedures, including tissue biopsies and fluid aspirations, has markedly increased despite the advances in imaging techniques and serological investigations. In many liver diseases, liver biopsy is still a gold standard for diagnosis, prognostication and assistance in determining the treatment plan. The increasing popularity of these procedures stems in part from their less invasive nature, lower risk compared with surgery, high diagnostic accuracy and substantial cost-effectiveness (1-10).

Although liver biopsy is an easy procedure for hospitalized patients and outpatients, some complications or technical failures may occur in up to 5% of the patients, including pain requiring hospital admissions, bleeding requiring transfusion, bleeding requiring surgery, pneumothorax, failure to obtain tissue and obtaining other tissues (2, 4, 5, 8). However, attempts to increase safety and decrease complications of liver biopsy should be made (2, 5). There is also a statistically significant decrease in the complication rates of liver biopsies when performed under ultrasound guidance (4, 6, 11, 12).

## 2. Objectives

In this paper, we evaluate the complications and quality of biopsy specimens obtained by percutaneous liver biopsy using the tru-cut biopsy gun in patients with diffuse liver disease who were scheduled for treatment in our center.

## 3. Patients and Methods

### 3.1. Patient Selection

In this study, we retrospectively evaluated the US guided tru-cut biopsies of the liver of 1018 patients attending our clinic between October 2006 and July 2010. Patients followed up with a diagnosis of diffuse involvement of liver lesions were subjected to US guided percutaneous liver biopsy for evaluation of lesions severity and planning of the treatment regimen. All patients were informed about the procedure and the potential postprocedure complications before the biopsies. Written informed consent was obtained from all patients. All patients underwent routine coagulation tests and a complete blood count. A whole abdominal ultrasonography was performed before the biopsy to identify probable asymptomatic masses and to determine the anatomy of the liver and adjacent organs. Biopsy was performed in those patients with a hemoglobin (Hb) value less than 10.0 gr/dL, platelet count higher than 80.000/l, INR less than 1.5 and a PTT time of no longer than forty seconds.

### 3.2. Procedure of Biopsy

We used a tru-cut biopsy gun called Angiotech Pro-Mag model and its compatible biopsy needle (18 gauge×20 cm in size) with a 17 mm specimen length and a 22 mm needle movement distance. For all biopsies, TOSHIBA Nemio ultrasonography device with a 3.5-MHz convex probe was used. The sizes, number of the biopsy specimens and prediagnosis according to radiologic features were recorded and then they were sent to our pathology department for histopathological examination.

After the procedure, the vital signs of all patients were controlled for 2-4 hours for the probable complications. We admonished all the patients to return to the hospital within 30 minutes after the onset of any adverse symptoms. The biopsy specimens were placed in 10% formaldehyde solution and transferred to our pathology laboratory consecutively on the same day.

## 4. Results

Between October 2006 and July 2010, 1018 patients were enrolled. In 987 cases (98.3%), adequate tissue was reported as sufficient material by the pathology department. The etiology included chronic hepatitis B (60.6%), chronic hepatitis C (23.7%), autoimmune liver disease (7.6%) and steatohepatitis (6.2%) (Table 1). 

**Table 1.** Analysis of the Patients

Type of Pathology	Patients,No (%)
**Chronic hepatitis B**	617 (60.6)
**Chronic hepatitis C**	242 (23.8)
**Autoimmune liver disease**	78 (7.7)
**Steatohepatitis**	64 (6.3)
**Insufficient material**	17 (1.6)
**Total**	1018 (100)

No biopsy-related death occurred in any of the patients. The most common complications were localized pain in 25 patients (80.6%) and vasovagal syncope or nausea in six patients (19.4%). The most common type was mild pain around the biopsy area that developed three hours after the biopsy. Other early complications were abdominal pain spreading to the right shoulder-arm. Interestingly, almost all vasovagal syncopes occurred during preparation, prior to biopsy.

## 5. Discussion

Inspite of the developed laboratory and imaging methods, the importance of liver biopsy in determining the true pathology is indisputable. Nevertheless, it has major disadvantages; it is an invasive method with a risk of complication and there are difficulties in evaluating the biopsy specimen. Biopsy of the liver under US guidance increases the diagnostic yield by obtaining sufficient liver specimen, subsequently increasing the probability of definitive pathological diagnosis (1, 2, 4, 5, 10, 13). In our series, the overall rates of complications were only 3% and most of them (80.6%) were minor and self-limited. The pain relieved rapidly with no need of analgesia. There was no mortality in the patient population. According to the findings of the wide-scale investigations, the average incidences of mortality and the complications of this procedure are 0.01% and 0.06-0.32%, respectively. In the literature, the US-guided percutaneous liver biopsy reduced post-biopsy pain significantly as well as the need of analgesics. Literature determined that the post-biopsy pain rates are between 5% and 50% (2, 4, 5, 8). Our study showed a lower pain and complication rate compared to those reported in the literature.

Even though the liver has a rich vascular supply, complications associated with percutaneous liver biopsy are very rare if the procedure is guided by US. The most frequent major complications of liver biopsy are hemorrhage and biliary leakage. Other complications include hemobilia, organ injuries, arterio-venous fistula and septic shock. Piccinino et al. (14) investigated 68276 biopsies over 10 years and the major complications were shock, pneumothorax, hemoperitoneum and biliary peritonitis (14). In our study, no major complication occurred. In accordance with our study, the most frequent complaint after biopsy was pain at the biopsy site and/or pain over the right shoulder probably due to irritation of the right hemidiaphragm.

According to the literature, cutting needles such as tru-cut needles are associated with a higher complication risk compared to that of aspirating needles because they remain longer inside the liver during the procedure and increase the risk of complications (15). However, as our study confirmed, US-guided liver biopsy is very safe and effective when carried out by the right hands.

Some studies recommend that the specimen should have a minimum length of 15 mm and should be composed of four to six portal areas; however, other studies suggest that the ideal specimen size should be at least 40 mm in length, and composed of at least two pieces with a minimum of 8 portal areas in each piece (16, 17). In our study, the average biopsy specimen size was 17 mm and comprised a minimum of 8 portal areas on each piece. Another significant issue is the location of the biopsy, as mentioned. If the biopsy specimen was less than 0.5cm or a necrotic core was obtained, the biopsy was repeated (1, 2, 18).

In conclusion, outpatient US-guided percutaneous liver biopsy with tru-cut biopsy needle is a very effective, safe and cost-effective procedure that is principally performed by radiologists. The percutaneous US-guided liver biopsy modality should be applied to all indicated cases by the right hands.
